# Fabricating nanopores with diameters of sub-1 nm to 3 nm using multilevel pulse-voltage injection

**DOI:** 10.1038/srep05000

**Published:** 2014-05-21

**Authors:** Itaru Yanagi, Rena Akahori, Toshiyuki Hatano, Ken-ichi Takeda

**Affiliations:** 1 Hitachi Ltd., Central Research Laboratory, 1-280 Higashi-koigakubo, Kokubunji, Tokyo, 185-8603

## Abstract

To date, solid-state nanopores have been fabricated primarily through a focused-electronic beam via TEM. For mass production, however, a TEM beam is not suitable and an alternative fabrication method is required. Recently, a simple method for fabricating solid-state nanopores was reported by Kwok, H. *et al*. and used to fabricate a nanopore (down to 2 nm in size) in a membrane via dielectric breakdown. In the present study, to fabricate smaller nanopores stably—specifically with a diameter of 1 to 2 nm (which is an essential size for identifying each nucleotide)—via dielectric breakdown, a technique called “multilevel pulse-voltage injection” (MPVI) is proposed and evaluated. MPVI can generate nanopores with diameters of sub-1 nm in a 10-nm-thick Si_3_N_4_ membrane with a probability of 90%. The generated nanopores can be widened to the desired size (as high as 3 nm in diameter) with sub-nanometre precision, and the mean effective thickness of the fabricated nanopores was 3.7 nm.

Recently, “nanopore” technology has been attracting great attention and has become an important subject for study because of its potential to achieve label-free single-molecule DNA sequencing (i.e., direct DNA sequencing) with very high throughput at low cost^1^,^2^,^3^,^4^,^5^,^6^,^7^,^8^,^9^,^10^,^11^,^12^,^13^,^14^,^15^,^16^,^17^,^18^,^19^,^20^,^21^,^22^,^23^,^24^,^25^,^26^,^27^. In addition to this advantage, another advantage, specifically, the potential to read long DNA sequences (i.e., long-read DNA sequencing), is given by direct DNA sequencing utilising nanopores. This advantage will enable the investigation of many unknown DNA markers derived from phase information^28^. These features of direct DNA sequencing are essential for providing personalised medicine in the future; therefore, the maturation of nanopore technology has been strongly anticipated.

Nanopore technology can be broadly divided into two categories according to the constituent materials of the nanopore. One is “biological”, i.e., nanopores that are formed with biological molecules (“bio-nanopores”)^1^,^2^,^3^,^4^,^5^,^6^,^7^. The other is “solid-state”, i.e., nanopores that are formed with semiconductor-related materials (“solid-state nanopores”)^1^,^7^,^8^,^9^,^10^,^11^,^12^,^13^,^14^,^15^,^16^,^17^,^18^,^19^,^20^,^21^,^22^,^23^,^24^,^25^,^26^,^27^. The most well-known concept of DNA sequencing, common to both bio-nanopores and solid-state nanopores, detects changes in the ionic current through the nanopore during DNA translocation and identifies the four types of nucleotides from the changes in ionic current^1^,^2^,^3^,^4^,^5^,^6^,^7^,^12^,^13^,^14^,^15^,^16^,^17^,^18^,^19^,^20^,^21^,^22^,^23^,^24^,^26^,^27^. To extract the ionic-current changes produced by the four types of nucleotides, the diameter of the nanopores must be on the order of that of DNA (less than 2 nm) because the differences in the molecular structures of each nucleotide are small. In addition to this requirement, to spatially discriminate each nucleotide in DNA, the thickness of the nanopores must be on the order of the distance between each nucleotide. To meet these requirements, both bio- and solid-state nanopore technologies have been developed.

In terms of biological nanopore technology, for example, the four types of nucleotides have been distinguished with high accuracies (averaging 99.8%) using an engineered α-haemolysin (α-HL) protein nanopore^2^. Moreover, according to a recent study, ionic-current patterns through a nanopore during DNA translocation have been related to a known sequence of nucleotides using an engineered mycobacterium smegmatis porin A (MspA) nanopore with a diameter of 1.2 nm and thickness of 0.6 nm^4^.

In terms of solid-state nanopore technology, poly(dA)_30_, poly(dC)_30_, and poly(dT)_30_ have been distinguished using a small nanopore (diameter of 1 to 2 nm) in a thin Si_3_N_4_ membrane (thickness of 5 to 8 nm)^12^. In addition, graphene nanopores have been developed with the aim of ultimate single-nucleotide resolution^18^,^19^,^20^,^21^,^22^,^23^, and DNA translocation through a graphene nanopore has been confirmed^18^,^19^,^20^,^21^.

Solid-state nanopore technology has advantages in terms of robustness and possible large-scale integration. However, this technology suffers a serious drawback regarding the process of fabricating the nanopores. To date, focused-electron beam etching via TEM has been used to fabricate nanopores in solid-state membranes. A TEM beam can be condensed to a diameter of less than 1 nm and can thereby be used to successfully fabricate a small nanopore with a diameter of less than 2 nm^12^,^14^,^24^,^25^. For mass production, however, TEM-beam etching is not suitable because of its high cost, low throughput, and complexity. An alternative nanopore fabrication method is therefore strongly desired.

Recently, a simple method for fabricating nanopores was proposed by Kwok *et al*.^26^. This method utilises the dielectric breakdown of a Si_3_N_4_ membrane in an aqueous solution to fabricate nanopores. The dielectric breakdown is caused by the strong electric field produced by two conventional Ag/AgCl electrodes, and no special setup for fabricating the nanopores is required. Nanopores with sizes as small as 2 nm were generated by the application of a high constant voltage (with a pre-determined threshold current level) to the electrodes, and the generated nanopores can be widened to the intended size by the application of pulse voltages. Ionic-current blockades were observed when double-stranded DNA (dsDNA) passed through the fabricated nanopores, and the ionic current exhibited a lower noise level compared with that of the current passing through conventional TEM-drilled nanopores. Accordingly, this fabrication method has the potential to open solid-state nanopore technology to a much greater number of researchers.

In this study, to stably fabricate nanopores with diameters of 1 to 2 nm (which is an essential size for distinguishing each nucleotide) via dielectric breakdown, a technique called “multilevel pulse-voltage injection” (MPVI) is proposed and demonstrated. Compared with Kwok's method, MPVI uses pulse voltages for not only widening the nanopores but also for creating the nanopores, and the generation of the nanopores is verified by measuring the current through a membrane at low voltage. This method can generate nanopores with diameters of less than 1 nm in a 10-nm-thick Si_3_N_4_ membrane with a probability of 90%. The diameter of the generated nanopores can be widened to the desired diameters (up to 3 nm) with sub-nanometre precision. The mean effective thickness of the fabricated nanopores was 3.7 nm. These findings are derived from TEM images of the fabricated nanopores and analyses of ionic-current blockades during single-stranded DNA (ssDNA) translocation.

## Results

### Proposition and demonstration of MPVI

**Figure 1:**
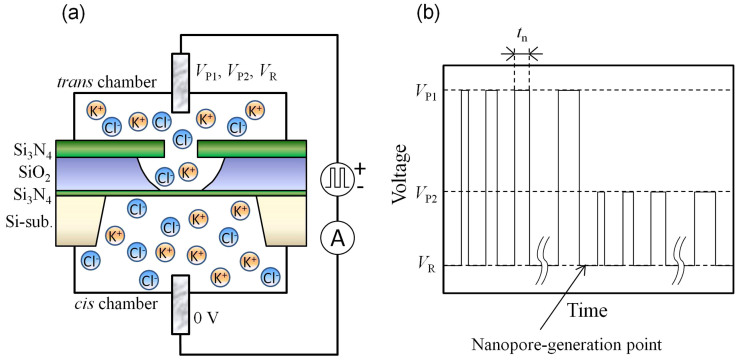
Schematic diagram of MPVI. (a) Setup for MPVI. *Cis* and *trans* electrodes are immersed in both chambers and are connected to a voltage-pulse generator and an ammeter. (b) Pulse chart of MPVI, which uses three different voltages (*V*_P1_, *V*_P2_, and *V*_R_). *V*_P1_ is used to create a nanopore. *V*_P2_ is used to widen the nanopore to an intended size. *V*_R_ is used to measure the current between the electrodes.

The setup for fabricating the nanopores by MPVI is illustrated in Figure 1a. Separated by a Si_3_N_4_ membrane with a thickness of 10 nm, two chambers (*cis* and *tran*s chambers) are formed in a flowcell. Both chambers are filled with 1 M KCl aqueous solution. Two Ag/AgCl electrodes (*cis* and *trans* electrodes) are immersed in aqueous solutions and connected to a pulse-voltage generator and an ammeter.

A pulse chart for MPVI is presented in Figure 1b. After a high-voltage pulse (*V*_P1_) is applied between the *cis* and *trans* electrodes to generate a nanopore in the membrane, an electrical current between the electrodes at a low voltage (*V*_R_) is measured to verify whether a nanopore is generated. If the measured current exceeds a pre-determined threshold current, it is judged that a nanopore has been generated. The nanopore-generation mechanism by MPVI is based on the dielectric breakdown induced by the high-electric-field stress, which is explained in detail by Kwok *et al*.^26^. These authors discussed the breakdown mechanism by investigating the creation process as a function of applied voltage, membrane thickness, electrolyte composition, concentration, and pH. After the nanopore has been generated, it can be slowly widened to the intended size via the application of mid-voltage pulses (*V*_P2_)^27^. The size of the nanopore can be estimated from the ionic current passing through it at low voltage (*V*_R_).

The benefits of MPVI are explained as follows. When a voltage is applied between the *cis* and *trans* electrodes, the total electrical current (*I*_Tot._) between the electrodes is 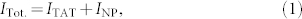
where *I*_TAT_ is the leakage current through the membrane and *I*_NP_ is the ionic current through the nanopore. *I*_TAT_ represents the non-ohmic characteristics and rapidly increases with increasing electric field strength^26^. This behaviour is similar to that of the trap-assisted tunnelling (TAT) current in semiconductor capacitors and transistors with Si_3_N_4_ gate dielectrics^29^,^30^,^31^. Accordingly, *I*_TAT_ is assumed to be primarily attributable to a TAT current due to electrons supplied from ions. In addition, *I*_TAT_ varies among several membranes with the same thickness and changes over time^26^. Thus, the correct value of *I*_NP_ cannot be measured because of a disturbance of the time-dependent *I*_TAT_ fluctuation if *I*_Tot._ is measured at a high voltage (*V*_P1_). If the correct *I*_NP_ cannot be measured, then a uniform threshold current value cannot be set to verify whether the nanopore is generated; i.e., if a uniform threshold current value is set, the small target size of the nanopore cannot be controlled.

In contrast to *I*_TAT_, *I*_NP_ represents ohmic characteristics^15^, and the condition *I*_NP_ ≫ *I*_TAT_ ~ 0 can be realised at a low voltage (*V*_R_). Accordingly, MPVI is an iteration sequence composed of applied high-voltage pulses (*V*_P1_) to generate or widen the nanopore and measurement of the electrical current between the electrodes at low voltage (*V*_R_).

**Figure 2:**
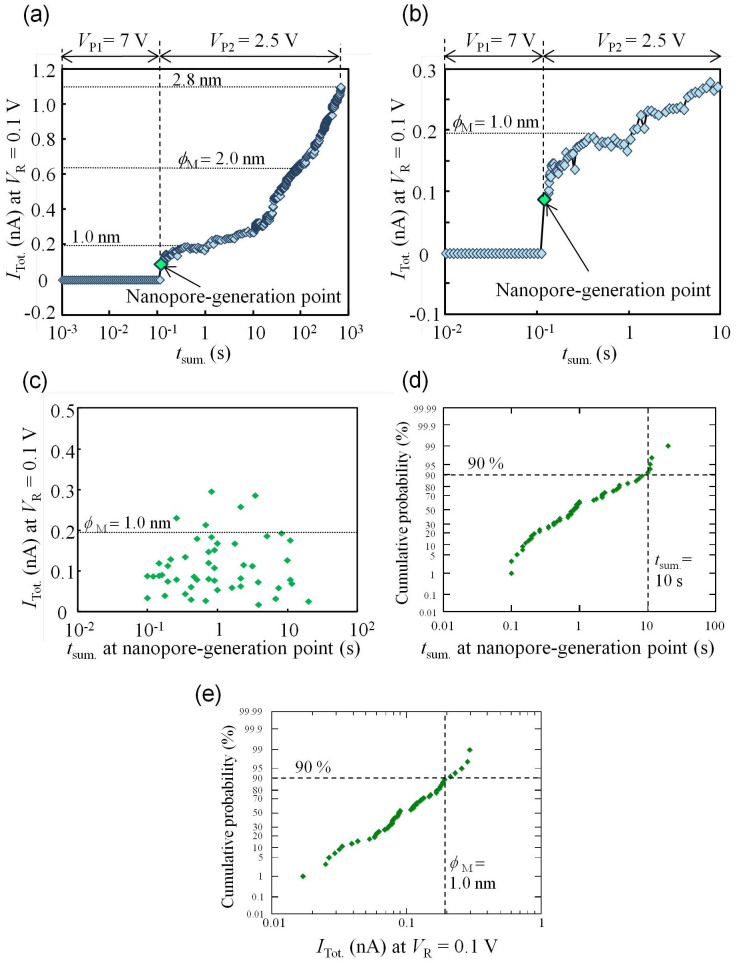
Time-dependent characteristics of current (*I*_Tot._) during MPVI. (a) Dependence of *I*_Tot._ at *V*_R_ = 0.1 V on the cumulative time of applied-pulse durations (*t*_sum._). A nanopore was generated at *t*_sum._ = 0.12 s. (b) Magnified graph around the nanopore-generation point. (c) Scatter plot of *I*_Tot._ at *V*_R_ = 0.1 V and *t*_sum._ at the nanopore-generation point. (d) Cumulative probability of *t*_sum._ at the nanopore-generation point. (e) Cumulative probability of *I*_Tot._ at the nanopore-generation point.

The dependence of *I*_Tot._ at *V*_R_ on the cumulated time (*t*_sum._ = Σ*t*_n_, where *t*_n_ is described below) of applied-pulse (*V*_P1_ and *V*_P2_) durations is shown in Figure 2a. *V*_P1_ was set to 7 V. The duration of the n_th_-pulse voltage (*V*_P1_) was set as 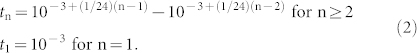
The number of the applied pulses (*V*_P1_) per decade of time was 24, and *V*_R_ was set to 0.1 V. More detailed information about the MPVI procedure is described in Supplementary Section SI-1. Before the nanopore was generated, *I*_Tot._ was approximately zero at *V*_R_. This result indicates that *I*_TAT_ can be neglected at *V*_R_ and that *I*_NP_ is free from disturbance by *I*_TAT_. After the nanopore was generated, second pulse voltages (*V*_P2_ = 2.5 V) widened its diameter. A magnified part of the graph around the nanopore-generation point in Figure 2a is shown in Figure 2b. This figure illustrates that the nanopore-generation point could be detected very easily and clearly because *I*_NP_ is free from the disturbance by *I*_TAT_.

**Figure 3:**
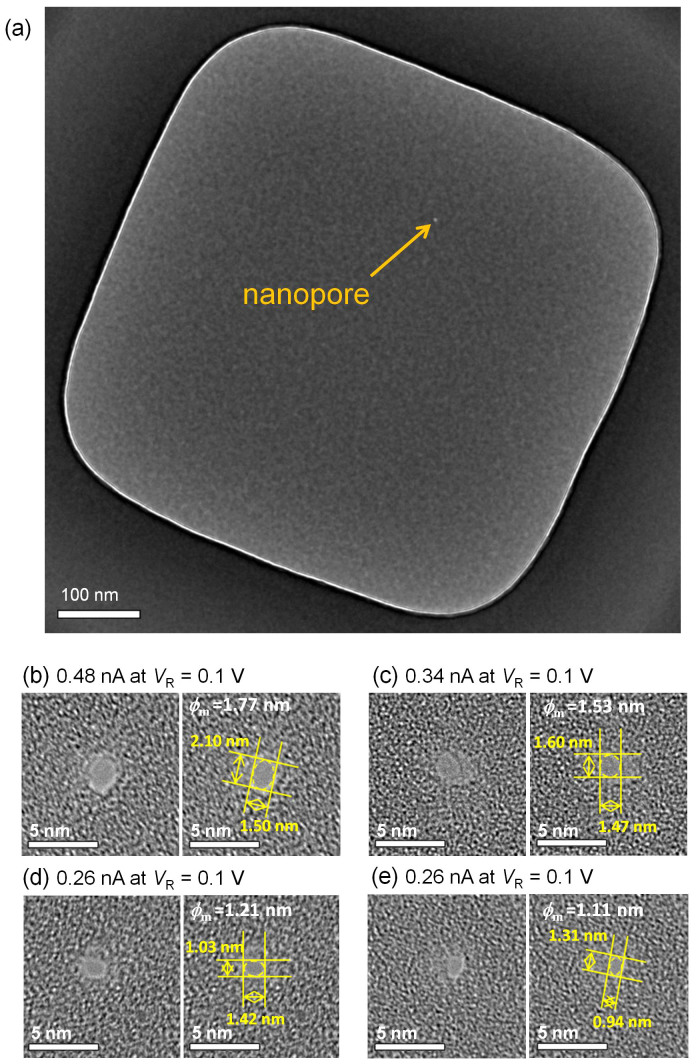
TEM images of nanopores fabricated via MPVI. (a) Top view of the entire area of the thinnest part of the membrane (approximately 500 × 500 nm^2^ square and 10-nm-thick Si_3_N_4_ membrane) with the nanopore. Figure (c) is a magnified view of the nanopore shown in (a). (b)–(e) Magnified views of the nanopores. Each left image shows the raw image of each right image. The ionic currents through the nanopore (*I*_Tot._ at *V*_R_ = 0.1 V) are (b) 0.48 nA, (c) 0.34 nA, (d) 0.26 nA, and (e) 0.25 nA.

**Figure 4:**
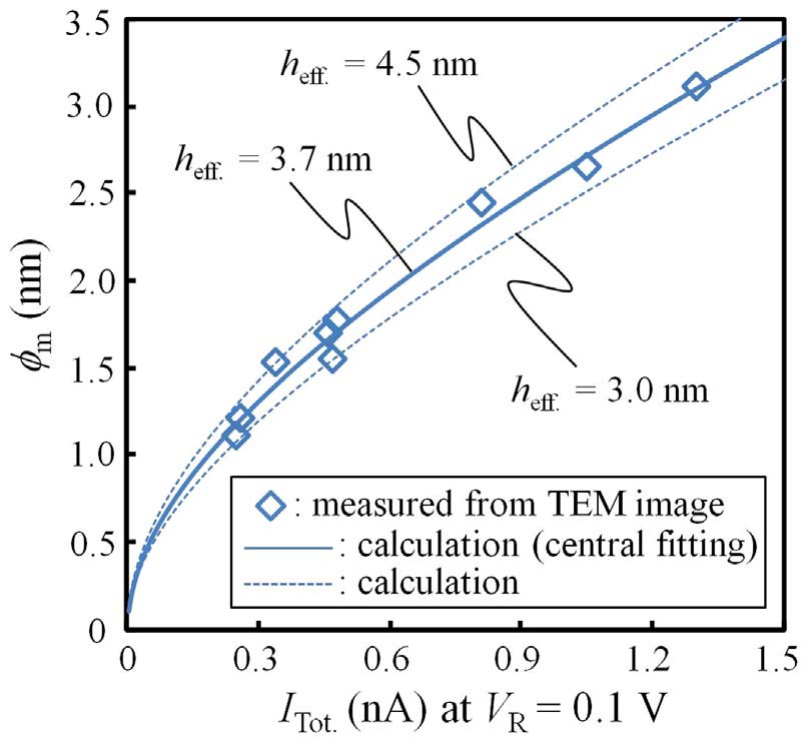
Relation between the mean diameters (*ϕ*_m_) of the fabricated nanopores and *I*_Tot._ at *V*_R_ = 0.1 V. Nine points are plotted within *ϕ*_m_ = 1.11 to 3.11 nm. The TEM images of all plotted points are presented in Figure 3 and Supplementary Section SI-2.

TEM images of the fabricated nanopores with MPVI are presented in Figure 3. Notably, in this work, all the nanopores were fabricated by MPVI with voltages set at *V*_P1_ = 7 V, *V*_P2_ = 2.5–3 V, and *V*_R_ = 0.1 V. The threshold current to verify the generation of the nanopore was set at 10 pA. These images confirm at a glance that only one nanopore was fabricated in the Si_3_N_4_ membrane (Figure 3a) and that nanopores with diameters less than 2 nm could be fabricated (Figures 3b–3e). Addition TEM images of nanopores larger (or smaller) than those observed in Figure 3 are presented in Supplementary Section SI-2, and the number of nanopores was one per membrane only. To determine the relation between the area of the nanopore and *I*_Tot._ at *V*_R_ = 0.1 V, the mean diameter of the nanopore (*ϕ*_m_) can be approximated by an ellipsoidal approximation as 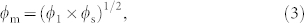
where *ϕ*_l_ and *ϕ*_s_ are the major and minor axes, respectively, of the nanopore measured from the TEM image. The dependence of *ϕ*_m._ on *I*_Tot._ at *V*_R_ = 0.1 V is illustrated in Figure 4. This figure shows that the mean diameter of the nanopore (down to 1 nm) can be precisely estimated by measuring *I*_Tot._ at *V*_R_ = 0.1 V. The plotted measurements agree well with the theoretically calculated lines obtained as follows^13^,^32^: 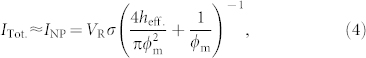
where *h*_eff_ is the effective height of the nanopore and σ = 0.105 S/cm is the measured conductance of the KCl buffer solution at 22.5°C. The calculation with *h*_eff_ of 3.7 nm is the central fitting line, and the variation in *h*_eff_ is small, within 3 to 4.5 nm. Accordingly, in this work, the mean diameters of the fabricated nanopores that were not observed by TEM were calculated from Equation (4) with *h*_eff_ = 3.7 nm and the measured *I*_Tot_. To discriminate the calculated mean diameter from the mean diameter (*ϕ*_m_) determined from the TEM images and Equation (3), another parameter, *ϕ*_M_ (which represents the diameter calculated from Equation (4) with *h*_eff_ = 3.7 nm and measured *I*_Tot._), is introduced in the following. Notably, the mean *h*_eff_ of the nanopores fabricated with MPVI is approximately one-third of the actual membrane thickness (10 nm) and the mean *h*_eff_ of the nanopores generated by the TEM beam is also approximately one-third of the actual membrane thickness^12^,^13^.

The characteristics observed in Figure 2 are reviewed in terms of the diameter *ϕ*_M_ as follows. A nanopore with *ϕ*_M_ of less than 1 nm could be generated and detected (as observed in Figures 2a and 2b). In addition, the generated nanopore could be widened to the intended size with sub-nanometre precision. A scatter plot of the cumulative pulse-duration time (*t*_sum._) and *I*_Tot._ (~*I*_NP_) at the nanopore-generation point (50 points are plotted) is presented in Figure 2c, and the cumulative probabilities of *t*_sum._ and *I*_Tot._ at the nanopore-generation point are plotted in Figures 2d and 2e, respectively. These figures illustrate that nanopores were rapidly generated within approximately *t*_sum._ ≤ 10 s and that nanopores with diameters of less than 1 nm could be generated with a probability of 90%. These data demonstrate that nanopores with diameters of <1 nm to 3 nm can be fabricated with sub-nanometre precision using MPVI.

### ssDNA translocation through the nanopore

**Figure 5:**
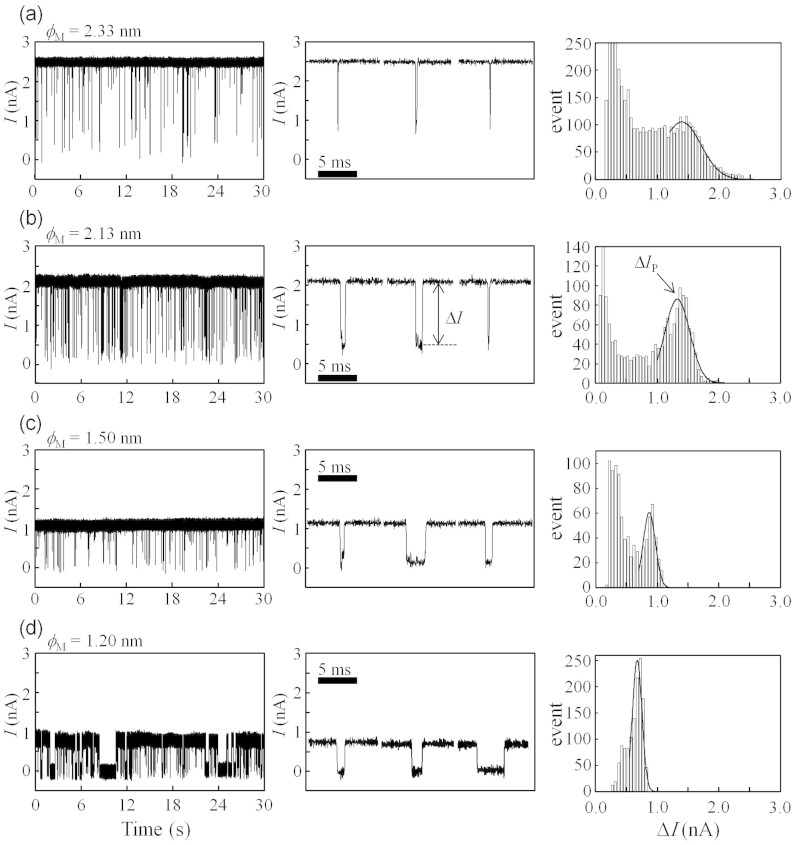
Detections of ssDNA translocations through nanopores with different *ϕ*_M_. The left figures show time traces of the ionic currents at 0.3 V. The middle figures are magnified views of the ionic-current blockades shown in the left figures. The right figures present histograms of the blockade currents (Δ*I*) with Gaussian-fit lines. Δ*I*_p_ was determined by the peak value of the Gaussian fit. Each data point was low-pass filtered at 10 kHz.

After the nanopore was fabricated using MPVI, the solution in the *cis* chamber was displaced by a 1 M KCl buffer solution with 1 nM 5.3-kb ss-poly(dA) without exposing the Si_3_N_4_ membrane to air. Details of the synthesis of 5.3-kb ss-poly(dA) are provided in the “Method” section. The ionic current through the nanopore was then measured at a voltage of 0.3 V (Figure 5). In the figure, ionic current blockades (Δ*I*) for each nanopore are observed, and each histogram of Δ*I* contains a discriminative peak (i.e., Δ*I*_P_, calculated from Gaussian fits to each histogram), indicating ssDNA translocations through the nanopore. As *ϕ*_M_ decreased, the dwell times of ssDNA in the nanopore became long because of increases in the interactions between ssDNA and the nanopore^16^.

When *ϕ*_M_ ≥ 1.2 nm, ssDNA-translocation events could be detected. When *ϕ*_M_ < 1.2 nm, the nanopore was clogged with ssDNA, and stable translocation events could not be detected (Supplementary Section SI-3). This threshold diameter is almost equal to the value reported by Venta *et al*.^12^.

In addition, when *ϕ*_M_ < 2.0 nm, the frequency of ssDNA-translocation events strongly depended on the applied voltage. The frequency significantly decreased as the voltage was decreased from 0.3 to 0.1 V (see “Supplementary Section SI-4”); thus, statistical analysis of Δ*I* was difficult at 0.1 V. However, when *ϕ*_M_ > 2.5 nm, the discriminative peak did not appear in the histogram at 0.3 V, and Δ*I*_P_ was difficult to determine because the dwell times of ssDNA in the nanopore became shorter and the translocation events could not be measured accurately (see Supplementary Section SI-5). To detect the translocation events accurately with decreasing translocation speed of ssDNA, the translocation events were detected, and Δ*I*_P_ was calculated at 0.1 V for *ϕ*_M_ > 2.5 nm.

**Figure 6:**
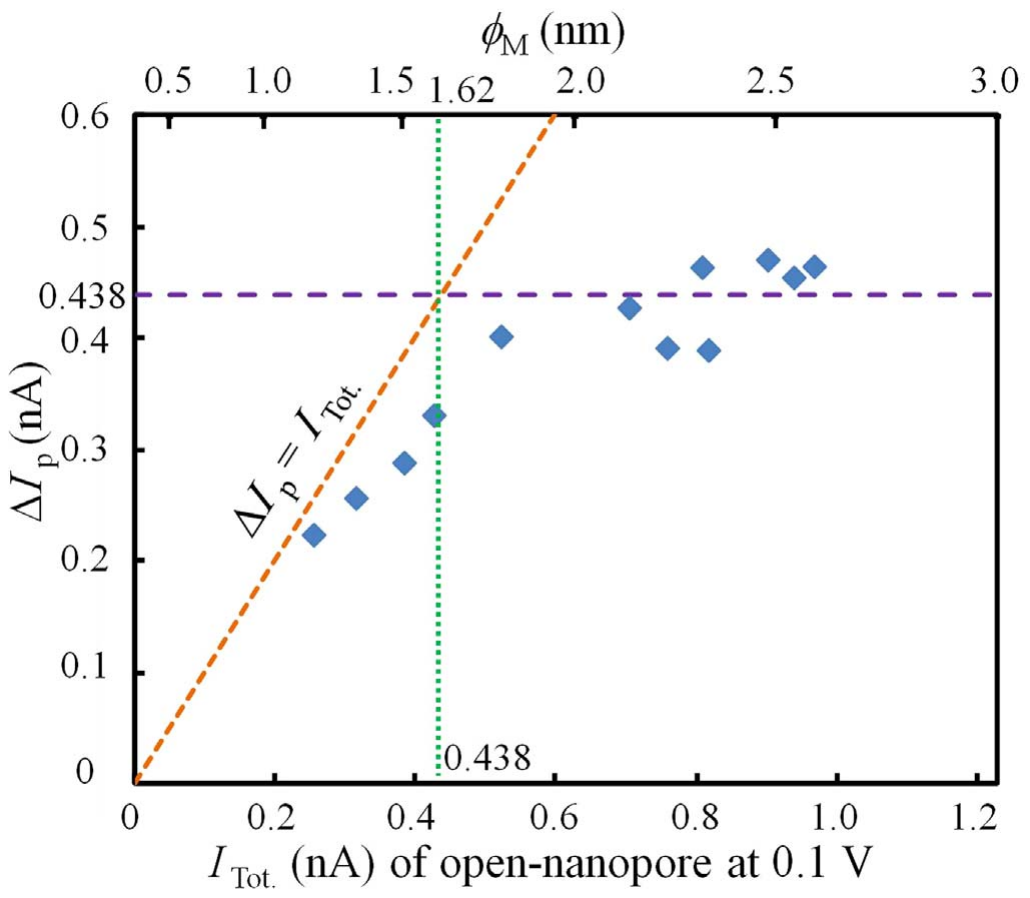
Dependence of Δ*I*_p_ on the *I*_Tot._ (nA) of open nanopores at 0.1 V. The purple horizontal line represents the average value of Δ*I*_p_ for *ϕ*_M_ > 2.1 nm (seven points). The sloping orange line represents Δ*I*_p_ = *I*_Tot._ (which represents an asymptote of the plots when *ϕ*_M_ < 1.62 nm).

The dependence of Δ*I*_P_ on *I*_Tot._ (open-nanopore current) is illustrated in Figure 6. Δ*I*_P_ and *I*_Tot._ were normalised at 0.1 V; i.e., Δ*I*_P_ and *I*_Tot._ at 0.3 V and *ϕ*_M_ < 2.5 nm were measured and divided by three (because Δ*I*_P_ and *I*_Tot. _are proportional to the applied voltage, as explained in Supplementary Section SI-4). When *I*_Tot._ was large (i.e., greater than 0.7 nA; *ϕ*_M_ > 2.1 nm, which is sufficiently larger than the diameter of ssDNA), Δ*I*_P_ was almost constant; i.e., the range of Δ*I*_P_ was 0.39 to 0.47 nA and the average value of Δ*I*_P_ was 0.44 nA, which corresponds to a *ϕ*_M_ of 1.62 nm. This value is reasonable because a *ϕ*_M_ of 1.62 nm fairly closely agrees with the diameter of ssDNA (approximately 1.4 nm). When *ϕ*_M_ < 1.62 nm, the value of Δ*I*_P_/*I*_Tot._ approached one and Δ*I*_P_ tended to be constrained by *I*_Tot._, suggesting that the diameter of ssDNA might decrease to fit the diameter of the given nanopore.

## Discussion

The proposed technique, called multilevel pulse-voltage injection (MPVI), was demonstrated to precisely and simply fabricate nanopores with diameters of 1 to 2 nm, which are essential sizes for distinguishing each nucleotide. MPVI is an iteration sequence that involves of the application high-voltage pulses (*V*_P1_) to generate or widen a nanopore and measurement of an electrical current between the electrodes (*I*_Tot._) at low voltage (*V*_R_) to verify whether the nanopore has been generated. When *V*_P1_ was set to 7 V, a nanopore could be rapidly generated (within approximately 10 seconds in the cumulated pulse-durations (*t*_sum._)) in a 10-nm-thick Si_3_N_4_ membrane. A nanopore could be generated with a diameter of less than 1 nm with a probability of 90% when a set of narrow-width pulses were used, i.e., then the number of the applied pulses per decade of time was 24. The diameter of the nanopore (down to sub-1 nm) could be estimated from *I*_Tot._ because the trap-assisted tunnelling current through the membrane could be ignored (*I*_TAT_ ~ 0) at low voltage (*V*_R_ = 0.1 V). After the nanopore was generated, its size could be adjusted to the desired size with sub-nanometre precision via the application of mid-voltage pulses (*V*_P2_ = 2.5–3 V). We thus concluded that, compared to the conventional technique of using a focused-electronic beam via TEM, MPVI enables the easy, rapid, and highly accurately fabrication of a nanopore. TEM observations of the nanopores fabricated by MPVI indicated that the relation between *I*_Tot._ and *ϕ*_m_ within a *ϕ*_m_ range of 1 to 3 nm was well explained theoretically, specifically, by Equation (4), with a mean *h*_eff_ = 3.7 nm. This result demonstrates that *ϕ*_m_ can be monitored with sub-nanometre precision through the measurement of *I*_Tot_. Moreover, ssDNA translocations through the nanopores fabricated by MPVI were successfully demonstrated. We observed that the value of Δ*I*_P_/*I*_Tot._ approached one when *ϕ*_M_ < 1.62 nm, which is approximately the same diameter as that of ssDNA. This result also indicates that nanopores with diameters of 1–2 nm can be precisely fabricated by MPVI. We thus conclude that MPVI is a promising approach for realising the practical use of solid-state nanopores.

## Methods

### Fabrication of membranes

First, 10-nm-thick Si_3_N_4_ membranes (with areas restricted within small (500 × 500 nm^2^) square areas such that the fabricated nanopore could be easily found) were prepared. The membranes were fabricated on an 8-inch silicon wafer with a thickness of 725 μm. First, a multilayer of Si_3_N_4_/SiO_2_/Si_3_N_4_ (12/250/100 nm) was deposited onto the front of the wafer, and a Si_3_N_4_ layer with a thickness of 112 nm was deposited onto the backside of the wafer. The top Si_3_N_4_ layer in each 500 × 500 nm^2^ square area and the backside Si_3_N_4_ layer in each corresponding 1038 × 1038 μm^2^ square area were subsequently etched by reactive-ion etching, followed by silicon-substrate etching with TMAH (tetramethylammonium hydroxide). The front surface of the wafer was coated with protective films (ProTEK®B3primer and ProTEK®B3, Brewer Science, Inc.) during the silicon-substrate etching. The protection film was removed by acetone after the silicon-substrate etching. Finally, the SiO_2_ layer in each 500 × 500 nm^2^ square area was removed with buffered hydrofluoric acid (BHF: HF:NH_4_F = 1:60 for 8 min) and thin-Si_3_N_4_-membrane portions with thicknesses of 10 nm were fabricated. The thickness of the bottom Si_3_N_4_ layer was reduced by 2 nm during the etching with BHF. Before the MPVI method was applied, the membranes were cleaned and hydrophilised on each side with argon/oxygen plasma (SAMCO, Inc., Japan) at 10 W, a flow rate of 20 sccm, and a pressure of 20 Pa for 45 s.

### Materials

Single-stranded poly(dA) was prepared by DNA synthesis reactions in two steps. In the first step, the reaction mixture contained 1 × buffer for KOD Plus Ver. 2, 0.2-mM dATP, 0.2-mM dTTP, 1.5-mM MgSO_4_, 0.3-μM dA20 primer (5′-(dA)_20_-3′), 0.3-μM dT45 primer (5′-(dT)_45_-3′), and 0.02-U/μL KOD Plus Ver. 2 polymerase (TOYOBO, Japan). The first reaction was performed using the following amplification process: denaturation at 94°C for 2 min, 45 cycles of denaturation at 94°C for 15 s, primer annealing at 60°C for 30 s, and extension at 68°C for 10 min. The reaction mixture was then purified with a QIAquick PCR purification kit (QIAGEN, Germany) and was subsequently dissolved in Buffer EB (5-mM Tris-HCl at pH 8*.*5) to obtain the first reaction product, i.e., complementary strands of poly(dA)-poly(dT) (ds-poly(dA)-poly(dT)). In the second step, the reaction mixture contained 1 × Ex Taq Buffer, 0.8-mM dATP, 0.5-μM dA20 primer, 5.0-ng/μL of the first reaction product, and 0.025-U/μL Ex Taq polymerase. The second reaction was performed using the following amplification process: denaturation at 94°C for 2 min, 45 cycles of denaturation at 94°C for 15 s, primer annealing at 30°C for 30 s, and extension at 72°C for 15 min. The reaction mixture was then purified with a QIAquick PCR purification kit and was dissolved in Buffer EB to obtain the second reaction product, i.e., ss-poly(dA). The lengths of the first (ds-poly(dA)-poly(dT)) and second (ss-poly(dA)) reaction products were estimated to be 5.5 ± 0.9 kbp and 5.3 ± 0.4 kb (average ± standard deviation), respectively, using alkaline-agarose-gel electrophoresis. Further details about the evaluation of the prepared ss-poly(dA) are provided elsewhere^33^.

### Observations of the fabricated nanopores by TEM

The fabricated nanopores were observed with a field-emission transmission electron microscope (JEM-2100F(HRP), 200 keV, JEOL, Ltd.). Before the observations, the membranes were immersed in warm DI water (32°C) for more than a day to remove any salt residues.

### Setup for MPVI and detection and analysis of ssDNA-translocation events

The prepared membrane was first mounted onto a custom-built acrylic flowcell. Separated by the membrane, two chambers (each with a volume of 90 μL) were formed in the flowcell: a *cis* chamber and a *trans* chamber. Both chambers were filled with a buffer solution consisting of 1 M potassium chloride, 10 mM Tris-HCl, and 1 mM EDTA buffer at pH 7.5. An Ag/AgCl electrode was immersed into each solution to assure electrical contact between the chambers.

The pulse voltages used in the MPVI were applied with a 41501B SMU AND Pulse Generator Expander (Agilent Technologies, Inc.), and the electrical currents between the electrodes were measured with a 4156B PRECISION SEMICONDUCTOR ANALYZER (Agilent Technologies, Inc.). The MPVI procedure was controlled by a programme written in Excel VBA (Visual Basic for Applications). Further information on the procedure is provided in Supplementary Section SI-1.

To detect ssDNA translocation events, a patch-clamp amplifier (Axopatch 200B, Axon Instruments, Union City, CA) was used to apply voltages and to detect the ionic current through the nanopores. The detected current was first low-pass filtered through a four-pole Bessel filter with a cutoff frequency of 10 kHz, then digitised with an NI USB-6281 18-bit DAQ AD converter (National Instruments, Austin, TX) at 50 kHz, and subsequently recorded onto the hard disk of a personal computer. Current-blockade events were identified and analysed using the Clampfit 10.2 software (Molecular Devices). The entire previously described procedure was performed at room temperature.

## Author Contributions

I.Y. developed the initial concept. I.Y. and R.A. proved the feasibility of the concept. I.Y. and R.A. designed and performed experiments and analysed the data. T.H. and R.A. prepared ssDNA. K.T. supervised the study.

## Supplementary Material

Supplementary Information Supplementary Information for Fabricating Nanopores with Diameters of Sub-1 nm to 3 nm Using Multilevel Pulse-voltage Injection
