# Supplementary material for: Fabricating nanopores with diameters of sub-1 nm to 3 nm using multilevel pulse-voltage injection
Source: Sci Rep. 2014 May 21;4:5000. doi: 10.1038/srep05000 (PMC4028839; doi:10.1038/srep05000)

Supplementary Information for
Fabricating Nanopores with Diameters of Sub-1 nm to 3 nm Using Multilevel Pulse-voltage Injection

Itaru Yanagi*, Rena Akahori, Toshiyuki Hatano, and Ken-ichi Takeda

Hitachi Ltd., Central Research Laboratory, 1-280 Higashi-Koigakubo, Kokubunji, Tokyo, 185-8603

The Supplementary Information includes:

SI-1. Detailed procedure and flowchart of MPVI

SI-2. Magnified TEM images of nanopores fabricated by MPVI

SI-3. Time trace of ionic current through a nanopore with ϕ_M = 0.88 nm

SI-4. Dependence of ssDNA-translocation-event frequency on applied voltage

SI-5. Dependence of ionic-current-blockade histogram on applied voltage

SI-1. Detailed procedure and flowchart of MPVI

The MPVI procedure is controlled by a programme written in Excel VBA (Visual Basic for Applications). The detailed flowchart of the programme is described below.

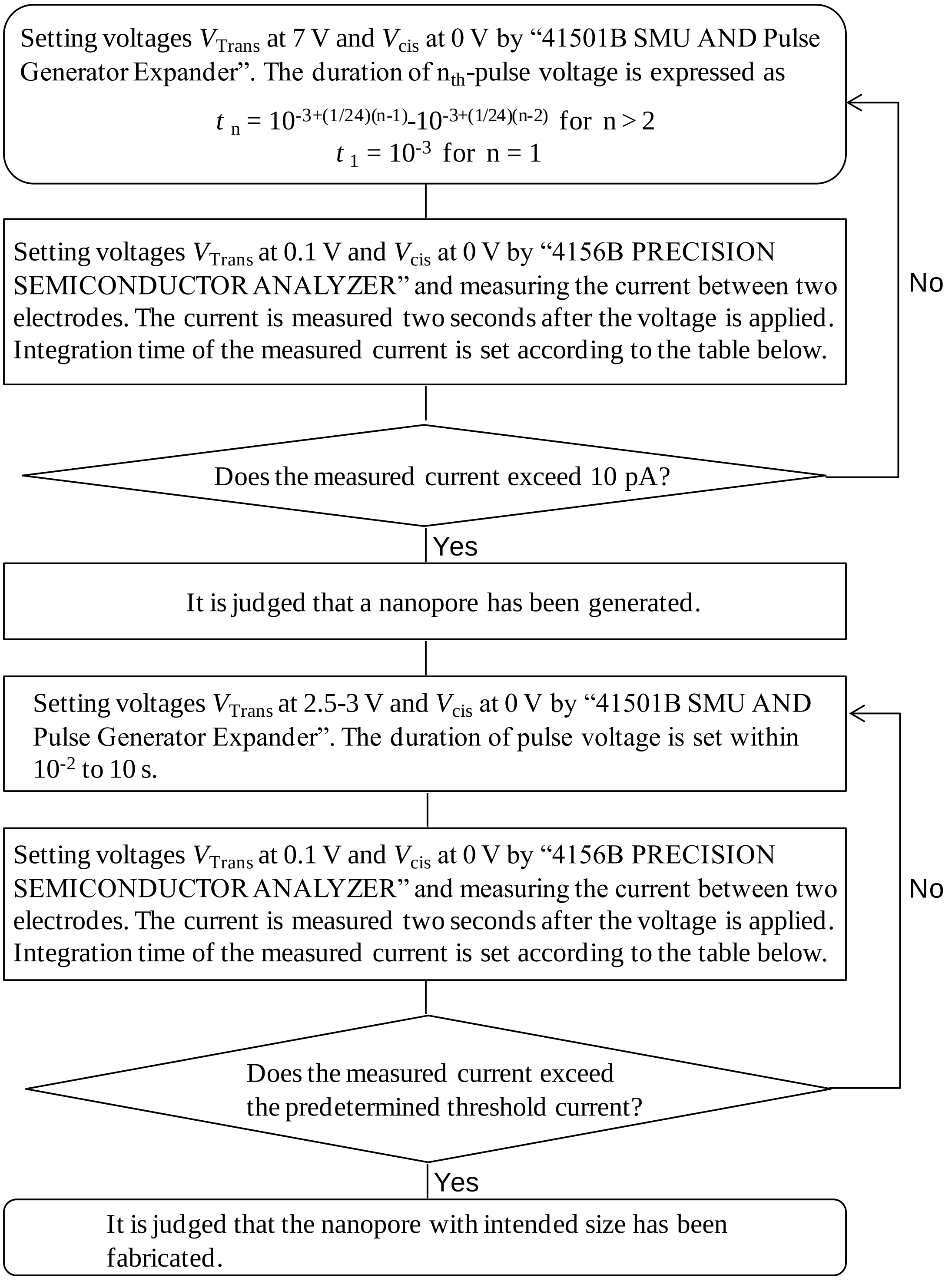

The integration time of the currents measured by a 4156B PRECISION SEMICONDUCTOR ANALYZER is listed in the table below. We allowed the integration time to be automatically changed according to the following table by setting it to “medium”.

measurement range	integration time
10 pA	1 s
100 pA	200 ms
1 nA	100 ms

SI-2. Magnified TEM images of nanopores fabricated by MPVI

Magnified views of the nanopores. Each left image shows the raw image of each right image. The ionic currents through the nanopore ($I_{Tot.}$ at $V_R = 0.1$ V) were (a) 1.30 nA, (b) 1.05 nA, (c) 0.81 nA, (d) 0.46 nA, (e) 0.47 nA, and (f) 0.08 nA. The nanopore with $I_{Tot.} = 0.08$ nA at $V_R = 0.1$ V was too small to measure.

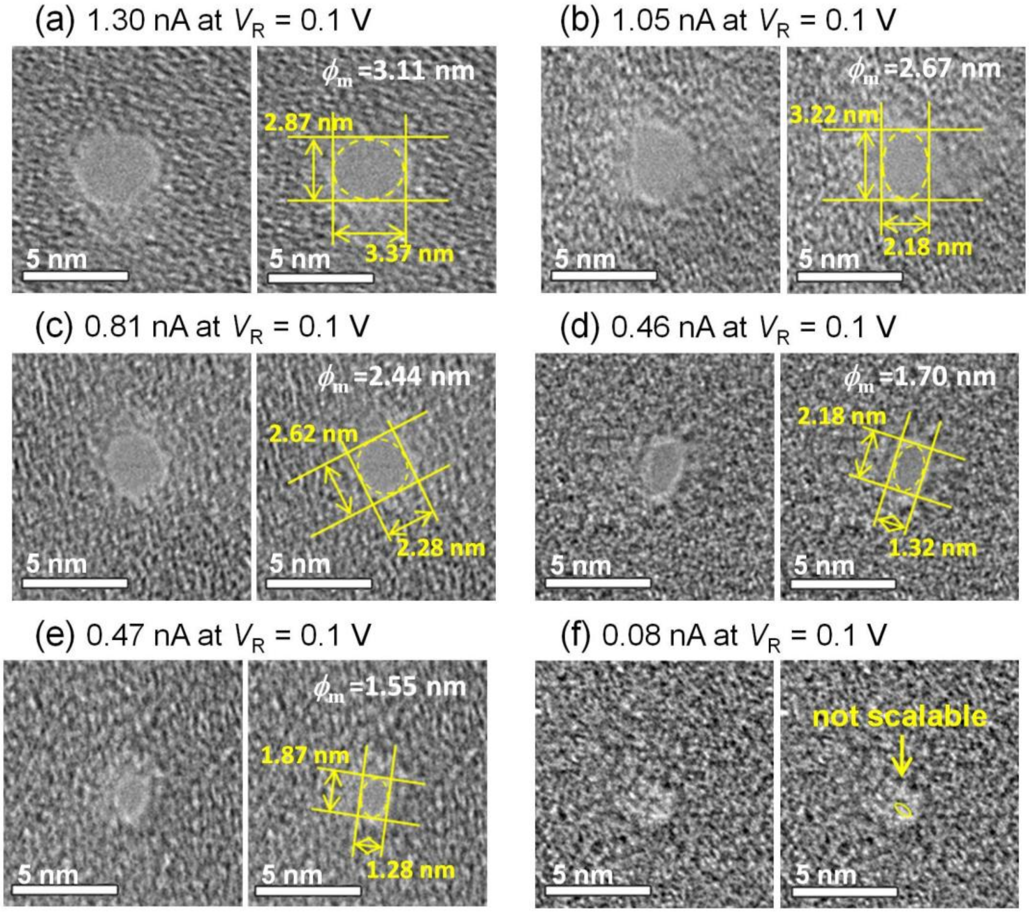

SI-3. Time trace of ionic current through a nanopore with ϕ_M = 0.88 nm

The ionic current was measured at 0.1 V, and the measurement data were low-pass filtered at 5 kHz. The nanopore was closed a few seconds after the start of the measurement, indicating that the nanopore was clogged with ssDNA. Although hyperpolarizing voltages (±1.3-V_{DC} pulse with a duration of 100 ms) were applied to remove ssDNA from the nanopore, the ionic current did not recover.

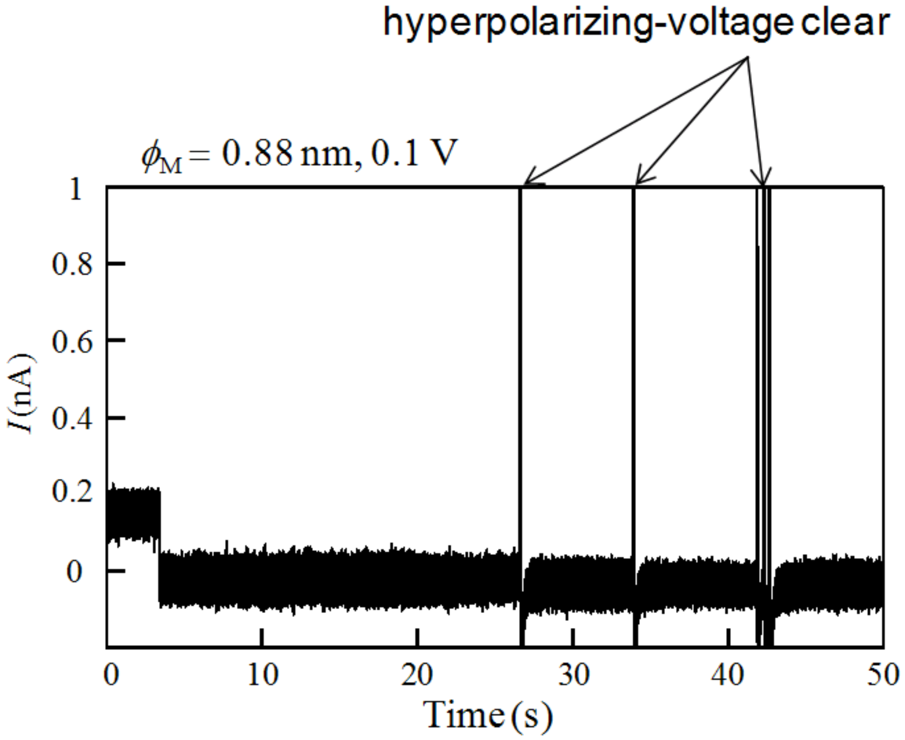

SI-4. Dependence of frequency of ssDNA translocation events on applied voltage

The ionic current was measured at (a) 0.3 V and (b) 0.1 V using the same nanopore with ϕ_M = 1.20 nm. Each data set was low-pass filtered at 5 kHz. The event frequency significantly decreased as the voltage was decreased from 0.3 to 0.1 V; the statistical analysis of ΔI was difficult at 0.1 V.

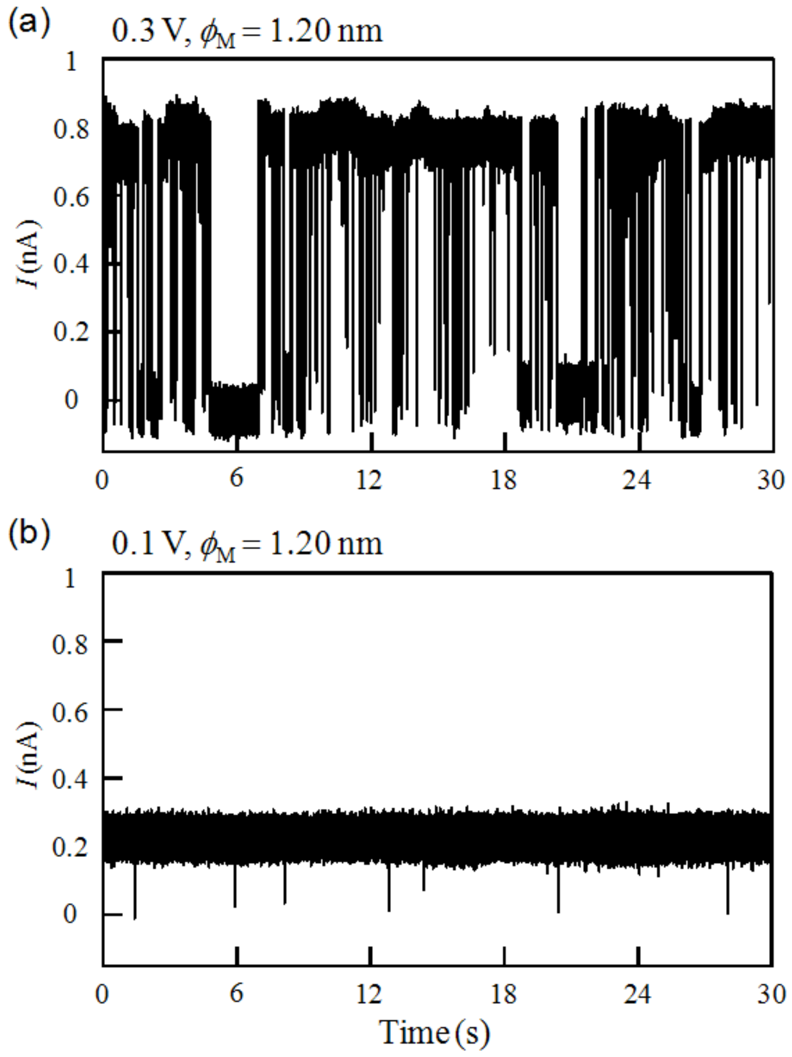

SI-5. Dependence of ionic-current-blockade histogram on applied voltage

The ionic currents were measured using the nanopores with ϕ_M = 2.33 nm (a-1 and a-2) and 2.55 nm (b-1 and b-2) at voltages of 0.3 V (a-1 and b-1) and 0.1 V (a-2 and b-2). Each data set was low-pass filtered at 10 kHz. ΔI_p was determined by the peak value of the Gaussian fit. In Figs. a-1 and a-2, a more discriminative peak in the histogram appears at 0.1 V. The ratio of ΔI_P at 0.3 V to ΔI_P at 0.1 V is approximately three. This result indicates that ΔI_P is proportional to the applied voltage. In Figs. b-1

and b-2, a discriminative peak in the histogram only appears at 0.1 V; it does not appear at 0.3 V. These results indicate that the translocation events cannot be measured accurately at 0.3 V when ϕ_M > 2.5 nm because the translocation speed of ssDNA through the nanopore increases with increasing ϕ_M.

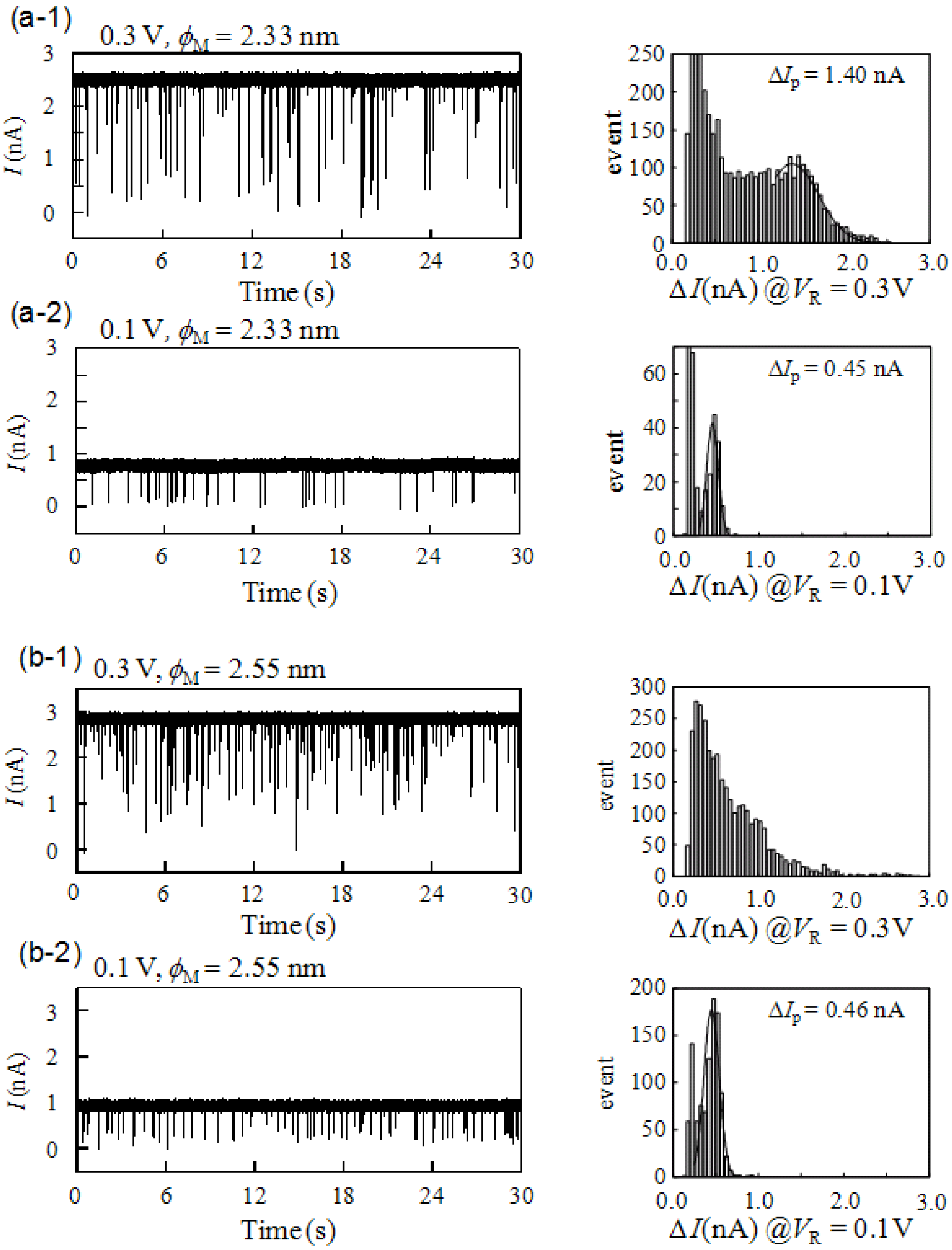

Supplement: Supplementary Information — for Fabricating Nanopores with Diameters of Sub-1 nm to 3 nm Using Multilevel Pulse-voltage Injection [file srep05000-s1.pdf]
